# The Effect of Long-Term Intranasal Serotonin Treatment on Metabolic Parameters and Hormonal Signaling in Rats with High-Fat Diet/Low-Dose Streptozotocin-Induced Type 2 Diabetes

**DOI:** 10.1155/2015/245459

**Published:** 2015-06-01

**Authors:** Kira V. Derkach, Vera M. Bondareva, Oxana V. Chistyakova, Lev M. Berstein, Alexander O. Shpakov

**Affiliations:** ^1^Laboratory of Molecular Endocrinology, Sechenov Institute of Evolutionary Physiology and Biochemistry, Russian Academy of Sciences, Thorez Avenue 44, Saint Petersburg 194223, Russia; ^2^Laboratory of Oncoendocrinology, N.N. Petrov Research Institute of Oncology, Leningradskaya Street 68, Pesochny, Saint Petersburg 197758, Russia

## Abstract

In the last years the treatment of type 2 diabetes mellitus (DM2) was carried out using regulators of the brain signaling systems. In DM2 the level of the brain serotonin is reduced. So far, the effect of the increase of the brain serotonin level on DM2-induced metabolic and hormonal abnormalities has been studied scarcely. The present work was undertaken with the aim of filling this gap. DM2 was induced in male rats by 150-day high-fat diet and the treatment with low dose of streptozotocin (25 mg/kg) on the 70th day of experiment. From the 90th day, diabetic rats received for two months intranasal serotonin (IS) at a daily dose of 20 *μ*g/rat. The IS treatment of diabetic rats decreased the body weight, and improved glucose tolerance, insulin-induced glucose utilization, and lipid metabolism. Besides, it restored hormonal regulation of adenylyl cyclase (AC) activity in the hypothalamus and normalized AC stimulation by *β*-adrenergic agonists in the myocardium. In nondiabetic rats the same treatment induced metabolic and hormonal alterations, some of which were similar to those in DM2 but expressed to a lesser extent. In conclusion, the elevation of the brain serotonin level may be regarded as an effective approach to treat DM2 and its complications.

## 1. Introduction

The type 2 diabetes mellitus (DM2) is a widespread noncommunicable disease (over 380 million patients worldwide). It is characterized by a pronounced insulin resistance, moderate hyperglycemia, hyperinsulinemia, dysfunctions of pancreatic *β*-cells, and impaired lipid metabolism. Functional and metabolic abnormalities provoked by DM2 have many severe complications, including the cardiovascular diseases, such as diabetic cardiomyopathy, diabetic angiopathy, hypertension, and atherosclerosis, which are considered to be the cause of death of circa 80% of diabetic patients [1, 2], and the CNS diseases, such as diabetic encephalopathy, cognitive deficiency, and neurological disorders [3, 4]. The main factors causing DM2-associated complications are insulin resistance as well as oxidative stress and endoplasmic reticulum stress giving rise to numerous cell damage, systemic dyslipidemia, lipotoxicity, and hyperhomocysteinemia [5, 6].

An important role in the etiology and pathogenesis of DM2 and its complications is ascribed to the changes in the signaling network in the central nervous system (CNS), in the hypothalamus in particular. These changes fall in two groups, primary and secondary. The former are a potential cause of impaired feeding behavior, metabolic abnormalities, and, finally, metabolic syndrome and DM2, and the latter lead to DM2 complications, including neurodegenerative and neuroendocrine diseases [7–9]. The restoration of functional activity of the brain signaling systems is a reliable way to prevent DM2 and its complications and to correct them. This refers to the signaling systems regulated by insulin, leptin, and the agonists of melanocortin (MCR), serotonin (5-hydroxytryptamine, 5-HTR), and dopamine (DAR) receptors, whose impaired activity is associated with hyperphagia, obesity, dyslipidemia, and insulin resistance typical of DM2 and metabolic syndrome [9–13]. The data are available demonstrating that MC_3_R and MC_4_R agonists and DA_2_R agonist bromocriptine improved insulin sensitivity and prevented the metabolic abnormalities in DM2 and metabolic syndrome [11, 14–18]. The treatment of diabetic patients with bromocriptine also reduced significantly the risk and severity of cardiovascular diseases, indicating that this drug possessing anti-Parkinsonism activity is able to prevent diabetic cardiomyopathy, atherosclerosis, and other severe cardiovascular complications of DM2 [19].

It was shown that the treatment of diabetic patients having depression and other neurological disorders with the selective serotonin reuptake inhibitors (SSRI) normalized the brain serotonin level, induced weight loss, decreased the level of glycosylated hemoglobin, and improved insulinemia and glucose tolerance [10, 20–22]. Along with this, SSRI ameliorated brain signaling and restored cognitive functions impaired in DM2 [23]. A great contribution to the antidiabetic effect of brain serotonin is its influence on the appetite and food intake via acting upon CNS motivational circuitry [24–26] and its prevention effect on insulin resistance and metabolic dysfunctions due to the influence of serotonin on hypothalamic proopiomelanocortin neurons [7, 27, 28]. The level of serotonin in the brain will be increased if it is delivered by intracerebral or intranasal route. Intranasal administration is more preferable because it is not traumatic, requires no special equipment, and is easily reproduced. The intranasal delivery of a variety of hormones, neuropeptides, and drugs is an efficient method of their administration into CNS and a promising therapeutic strategy in the treatment of metabolic disorders and neurodegenerative and neurological diseases [29]. It was demonstrated in our earlier experiments that the long-term treatment of female rats with the neonatal model of DM, using intranasal serotonin (IS) administration, resulted in the improvement of glucose tolerance, spatial memory, and learning capacity and in the partial restoration of hormonal signaling in diabetic cohort of animals [13]. However, so far no comprehensive study of the influence of long-term IS treatment on glucose homeostasis, insulin resistance, lipid metabolism, and hormonal signaling in the hypothalamus and myocardium in DM2 has been carried out.

An important role in the etiology and pathogenesis of DM2 and its complications is assigned to alterations in the brain and peripheral hormonal signaling systems, the hormone-sensitive adenylyl cyclase signaling system (ACSS) in particular [30–32]. The ACSS plays a key role in the regulation of contractile function of the heart by adrenergic agonists. The latter specifically interact with different types of adrenergic receptors (AR), which leads to the activation of heterotrimeric G-proteins of the stimulatory (G_s_) or inhibitory (G_i_) types and induces the stimulation or inhibition of the enzyme adenylyl cyclase (AC), respectively. Many neurotransmitters and neurohormones also exert regulatory effects on the biochemical and physiological processes in the CNS and in the periphery via ACSS. It was shown by us and the other authors that the changes in ACSS activity in diabetic tissues correlate positively with severity and duration of DM2 [30–36]. Therefore, the study of ACSS is one of the most promising approaches to be used in the case of DM2 for identification of functional abnormalities in the CNS and the periphery as well as for monitoring dysfunctions in the nervous, cardiovascular, and other systems and for evaluation of the effectiveness of antidiabetic therapy [13, 31, 32, 37].

The aim of this work was to study the influence of long-term treatment of diabetic male rats with intranasally administered serotonin on glucose tolerance, insulin-induced glucose utilization, lipid metabolism, and functional activity of the hormone-sensitive ACSS in the hypothalamus and myocardium. The effect of long-term IS treatment on the analogous indications in nondiabetic animals was studied in parallel. The model of DM2 was achieved by 5-month high-fat diet (HFD) and the treatment of obese animals (on the 70th day of experiment) with a low dose of streptozotocin (STZ). This model, being the most suitable animal model of severe DM2, has been widely used in the recent years [38, 39]. The hypothalamus was chosen for AC experiments owing to the involvement of the AC signaling pathways in the regulatory effects of such hypothalamic regulators as serotonin, dopamine, and melanocortins [40, 41]. The choice of myocardial ACSS sensitive to adrenergic agonists had two reasons: first, cardiovascular diseases are most common and socially important complications of DM2, and second, the hypothalamic serotonin signaling impaired in DM2 is involved in the regulation of the sympathetic nervous system and, thus, controls the functions of the cardiovascular system [42–44]. In the present study intranasally administered serotonin is shown as having rather high efficiency in correcting impaired glucose tolerance and other metabolic abnormalities in HFD/low-dose STZ-induced DM2 as well as in improving AC signaling in the hypothalamus and myocardium significantly altered in diabetic pathology. This suggests a crucial role of the brain serotonin deficiency in the etiology and pathogenesis of DM2 and its complications. One of the promising ways to compensate for this deficiency is intranasal route of delivery of serotonin and its functional analogs.

## 2. **Material and Methods**

### 2.1. Animals

Adult male Wistar rats housed in plastic sawdust-covered cages with 6 animals in each cage with a normal light-dark cycle (12 h/12 h, light on at 09 h) and free access to food and water were used. Experiments were carried out under the Bioethics Committee of Sechenov Institute of Evolutionary Physiology and Biochemistry, Russian Acad. Sci., St. Petersburg, Russia (Institutional Guidelines, December 23, 2010), and according to “Guide for the Care and Use of Laboratory Animals” and to the European Communities Council Directive of 1986 (86/609/EEC) and all efforts were made to minimize animal suffering and to reduce the number of animals used.

### 2.2. The HFD/Low-Dose STZ Model of DM2 and the Treatment with Intranasally Administered Serotonin

**Figure 1:**
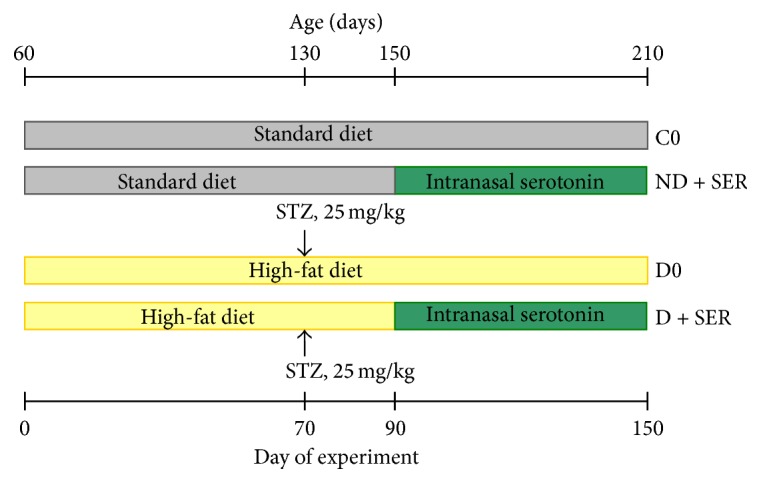
The design of HFD/low-dose STZ model of DM2 and the treatment of rats with intranasally administered serotonin.

After a one-week adaptation period, two-month-old rats were randomly divided into two groups, diabetic (*n* = 18, Group D) and control (*n* = 14, Group C). Diabetic rats received HFD, which included a prescribed amount of high-fat mixture (15 g/day/rat) and dry food given* ad libitum* (on average: 7–12 g/day/rat), while control animals received a standard diet, which included dry food given* ad libitum* (on average: 20–30 g/day/rat). One kilogram of the mixture consisted of 524 g of pork lard, 417 g of curd, 50 g of liver, 5.3 g of L-methionine, 1.85 g of baker's yeast, and 1.85 g of NaCl. Every day, accurately at 10.30, rats of Group D were fed the high-fat mixture. Seventy days after start of the experiment Group D was treated with freshly prepared STZ (“Sigma-Aldrich,” St. Louis, MO, USA) in 0.1 M citrate buffer, pH 4.5, which was administered intraperitoneally at a dose of 25 mg/kg of body weight. Control animals received buffer solution instead of STZ. Ninety days after the start of experiment Groups C and D were divided into two subgroups, Group C into C0 (*n* = 6) and ND + SER (*n* = 8) and Group D into D0 (*n* = 10) and D + SER (*n* = 8). Groups ND + SER and D + SER immediately began to receive a daily treatment with IS, whereas Groups C0 and D0 received placebo (Figure 1).

Serotonin at a final concentration of 1 mg/mL in 0.1 M citrate buffer, pH 4.5, was given intranasally for two months, 20 *μ*g per rat daily at 10.00. In Group D + SER, rats were treated by IS half an hour before being fed with a high-fat mixture. The daily dose of IS was taken according to the results of preliminary experiments and the data of the other authors who used intracerebral administration of serotonin and selective 5-HTR agonists [25, 45, 46] as well as the intranasal administration of the 5-HT_1_R agonist sumatriptan [47]. To do this, each rat was placed in a supine position and then an average of 20 *μ*L of serotonin solution containing 20 *μ*g of hormone was administered by Eppendorf pipette as 5 *μ*L drops in each nostril with 1-2 min interval. Control animals were given intranasally the same volume of buffer, pH 4.5. Control and diabetic animals were anesthetized and decapitated 150 days after the start of experiment. Decapitation of animals and the removal of the hypothalamus and myocardium covered the period from 10.00 to 11.00.

### 2.3. Plasma Glucose, Insulin, Triglycerides, and Cholesterol Measurements

Glucose in the whole blood from the tail vein was measured using a glucometer (“Life Scan Johnson & Johnson,” Denmark) and test strips “One Touch Ultra” (USA). Measuring of fasting glucose was made every 15 days; glucose concentration on the 60th, 90th, and 150th days of experiment is shown in Table 1. Insulin concentration in the rat serum was determined on the 60th and 150th days using Rat Insulin ELISA (“Mercodia AB,” Sweden). The concentrations of total triglycerides, total cholesterol, low-density lipoprotein (LDL), and high-density lipoprotein (HDL) cholesterols were measured on the 150th day of experiment using enzyme colorimetric kits obtained from “Olvex Diagnosticum” (Russia).

### 2.4. Glucose (GTT) and Insulin Glucose Tolerance (IGTT) Tests

Glucose tolerance test (GTT) was performed in the morning by i.p. injection of* D*-glucose at a single dose of 2 g/kg (“Sigma,” USA) after 12 h fasting on the 60th, 90th, and 147th days of experiment. Insulin glucose tolerance test (IGTT) was performed 10 days prior to decapitation (on the 140th day of experiment); to do this,* D*-glucose (2 g/kg, i.p.) and insulin (Humalog, 0.8 IU/kg b. w., s.c.) were administered simultaneously. Plasma samples were collected very fast before 0 min and 15, 30, 60, and 120 min after glucose (in GTT) or glucose plus insulin administration (in IGTT). The area under the curves (AUC) of glucose and insulin concentration from time 0 to 120 minutes (AUC_0–120_) was calculated.

### 2.5. Chemicals and Radiochemicals

Serotonin, noradrenaline, isoproterenol, (±)-(*R* ^*∗*^, *R* ^*∗*^)-[4-[2-[[2-(3-chlorophenyl)-2-hydroxyethyl]amino]propyl]phenoxy]acetic acid sodium hydrate (BRL-37344), 5-[(2*R*)-2-[[(2*R*)-2-(3-chlorophenyl)-2-hydroxyethyl]amino]propyl]-1,3-benzodioxole-2,2-dicarboxylate disodium hydrate (CL-316243), dopamine, 5-methoxy-*N*,*N*-dimethyltryptamine (5-MeO-DMT), bromocriptine, *α*-melanocyte-stimulating hormone (*α*-MSH), *γ*-MSH, somatostatin-14, pituitary adenylyl cyclase-activating polypeptide-38 (PACAP-38), relaxin, *β*,*γ*-imidoguanosine-5′-triphosphate (GppNHp), and forskolin were purchased from “Sigma-Aldrich” (St. Louis, MO, USA) while* N*-[(1*R*)-1-[(4-Chlorophenyl)methyl]-2-[4-cyclohexyl-4-(1*H*-1,2,4-trazol-1-ylmethyl)-1-piperidinyl]-2-oxoethyl]-1,2,3,4-tetrahydro-3-isoquinolinecarboxamide (THIQ), 5-nonyloxytryptamine (5-NOT), and 5-chloro-2-methyl-3-(1,2,3,6-tetrahydro-4-pyridinyl)-1*H*-indole (EMD-386088) were purchased from “Tocris Cookson Ltd.” (United Kingdom). [*α*-^32^P]-ATP (4 Ci/mmol) was obtained from “Isotope Company” (St. Petersburg, Russia).

### 2.6. Plasma Membrane Preparation

The preparation of synaptosomal membranes from rat hypothalamus was performed as described earlier [48]. The hypothalamus was separated from the other brain regions on ice and immediately homogenized with a Polytron in 30 volumes of ice-cold 50 mM Tris-HCl buffer (pH 7.4) containing 10 mM MgCl_2_, 2 mM EGTA, 10% (w/v) sucrose, and a cocktail of protease inhibitors including 500 *μ*M* O*-phenanthroline, 2 *μ*M pepstatin, and 1 mM phenylmethylsulfonyl fluoride (Buffer A). The obtained material underwent several centrifuge procedures, each performed at 4°C. Crude homogenate was centrifuged at 1000 ×g for 10 min; the resulting pellet was discarded and supernatant was centrifuged at 9 000 ×g for 20 min. The pellet was resuspended in Buffer A (without sucrose) and centrifuged finally at 35 000 ×g for 10 min.

The preparation of cardiac membranes from the rat myocardium was performed according to Baker and Potter [49], with some modifications [50]. The dissected hearts were placed in ice-cold 0.9% NaCl, and the atria, fat, and valves were removed. Then the obtained tissues were cut into small pieces, homogenized with a Polytron in 20 volumes of ice-cold 40 mM Tris-HCl buffer (pH 7.4) containing 5 mM MgCl_2_, 320 mM sucrose, and a cocktail of protease inhibitors (Buffer B), and centrifuged at 480 ×g for 10 min at 4°C. The pellet was discarded and supernatant centrifuged at 27 500 ×g for 20 min at 4°C. The pellet was resuspended in Buffer B (without sucrose) and then centrifuged at 27 500 ×g for 20 min.

The final pellet was resuspended in the 50 mM Tris-HCl buffer (pH 7.4) to obtain the membrane fraction with a protein concentration range of 1–3 mg/mL and stored at −70°C. Protein concentration in each membrane preparation was measured by the Lowry method using BSA as a standard [51].

### 2.7. Adenylyl Cyclase Assay

Adenylyl cyclase (EC 4.6.1.1) activity was measured as described earlier [52]. The reaction mixture (final volume 50 *μ*L) contained 50 mM Tris-HCl (pH 7.4), 5 mM MgCl_2_, 1 mM ATP, 0.5–1 *μ*Ci [*α*-^32^P]-ATP, 0.1 mM cAMP, 20 mM creatine phosphate, 0.2 mg/mL creatine phosphokinase, and 15–45 *μ*g of membrane protein. Incubation was carried out at 37°C for 12 min. The reaction was initiated by the addition of membrane protein and terminated by the addition of 100 *μ*L of 0.5 M HCl, followed by immersing the tubes with mixture in a boiling water bath for 6 min. 100 *μ*L of 1.5 M imidazole was added to each tube. In these conditions the AC activity was linear. [^32^P]-cAMP formed as a result of enzyme reaction was separated using column chromatography. The samples were placed on neutral alumina columns and cAMP was eluted with 12 mL of 10 mM imidazole-HCl buffer (pH 8.0). The eluate was collected in scintillation vials and was counted using a LKB 1209/1215 RackBeta scintillation counter (Sweden). Each assay was carried out in triplicate at least three times, and the results were expressed as pmol cAMP/min per mg of membrane protein. The basal activity was measured in the absence of hormonal and nonhormonal regulators of the enzyme. To measure the inhibiting effects of hormones, AC was preactivated by forskolin (10^−5^ M), five minutes before the addition of hormone.

### 2.8. Statistical Analysis

The animal groups were analyzed following mixed ANOVA, using two factors, including a group (Group), as between-group factor, and the time from the starting point of experiment (Time), as repeated measure within-group factor. The normality of distribution for all interventions at all time points was assessed by Shapiro-Wilk's test (*P* < 0.05). In case the interaction was statistically significant, the difference between groups at each level of Time was assessed, and the main simple effects were estimated, using one-way analysis of variance (ANOVA). If the interaction of factors was estimated as statistically nonsignificant, the main effects of two factors were estimated. In case when the difference between two single measurings was accessed, the* t*-test was performed. In each case the difference was considered significant at *P* < 0.05. The data are presented as the mean ± standard deviation. The difference in the AC activities in the membranes treated by hormones, forskolin, and GppNHp in different groups of rats was assessed statistically using the one-way ANOVA (*t*-test) and was considered significant at *P* < 0.05. The data are presented as M ± SD.

## 3. Results

### 3.1. The Food Consumption, Body Weight, Fasting Glucose Level, Glucose Tolerance and Utilization, and Lipid Metabolism in Rats with HFD/Low-Dose STZ DM2

**Figure 2:**
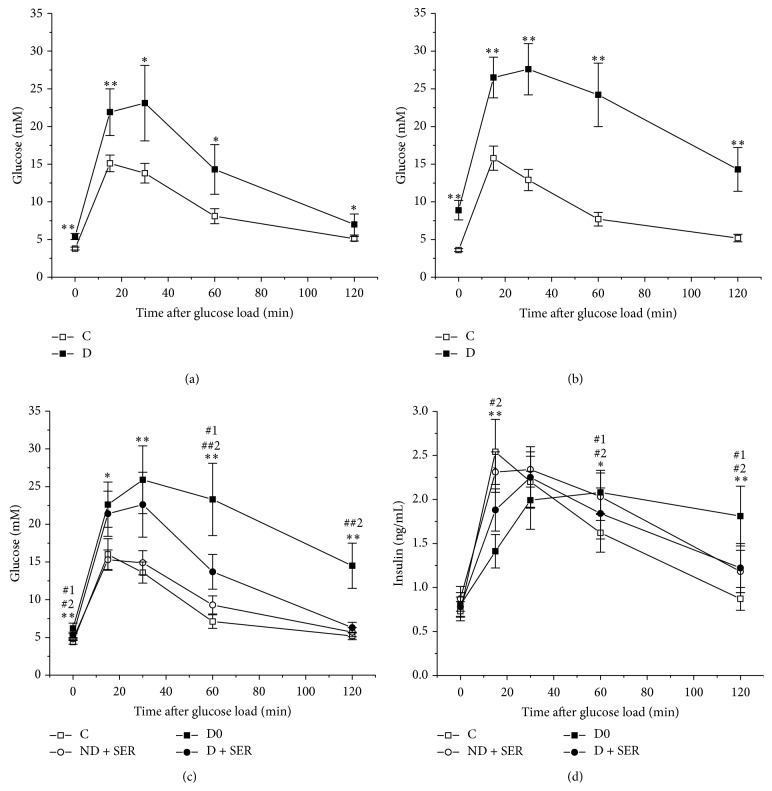
The concentration of plasma glucose in the glucose tolerance test in diabetic and nondiabetic rats on the 60th (a), 90th (b), and 147th days (c) and the concentration of insulin in glucose tolerance test on the 147th day (d) of experiment. In Figures 2(a) and 2(b): Group C (*n* = 7) and Group D (*n* = 9). In Figures 2(c) and 2(d): Group C (*n* = 6), Group ND + SER (*n* = 8), Group D0 (*n* = 10), and Group D + SER (*n* = 8). The data are presented as M ± SD. (*∗*, *∗∗*) The difference between Groups C and D (a, b) and between Groups C0 and D0 (c) and (#, ##) the difference between Groups C0 and ND + SER (1) or between Groups D0 and D + SER (2) are significant at *P* < 0.05 and *P* < 0.0001, respectively.

Prior to the start of the study, in the rats assigned to different experimental groups there was no difference in food intake, body weight, and metabolic parameters. In the first two months the food consumption in both groups, control and diabetic, did not differ significantly, and the animals consumed approximately 80 kcal/day/rat. The rats with HFD, unlike control animals, demonstrated a significant decrease in the total gram intake of the calorically dense diet, which led to normalization of the caloric consumption. Additionally, the average fat intake by the rats in Group D (~30 kcal/day/rat) was significantly higher as compared with control (~8 kcal/day/rat) and corresponded to the content of fat in the food. The increased fat consumption of rats in Group D caused weight gain and metabolic abnormalities characteristic of DM2 (Table 1 and Figure 2(a)). In Group D the body weight increased by 25% compared with Group C. Fasting glucose and insulin level increased by 38 and 27% (*t*-test, *P* < 0.01), demonstrating a decrease of insulin sensitivity. In Group D glucose level in GTT at all time points was significantly higher than in control (one-way ANOVA, *F*(1; 14) = 41.68,* P* = 1.5 *∗* 10^−5^, at 30 min, *F*(1; 14) = 74.29,* P* = 5.6 *∗* 10^−7^, at 60 min, and *F*(1; 14) = 89.65,* P* = 1.8 *∗* 10^−7^, at 120 min), and the AUC_0–120_ value for glucose concentration curve in Group D exceeded that in Group C by 61% (1744 ± 324 versus 1083 ± 65, *P* < 0.001). These results give the evidence in favor of the impaired glucose tolerance in rats on a two-month HFD.

Twenty days after the treatment with low-dose STZ (before the start of IS treatment) diabetic rats demonstrated an excess of body weight, hyperglycemia, and impaired glucose tolerance which surpassed the same indices in Group D on the 60th day of experiment (Table 1 and Figure 2(b)). AUC_0–120_ for glucose concentration during GTT in Group D exceeded that in the corresponding control group by 147% (2605 ± 389 versus 1055 ± 56, *P* < 0.0001).

**Figure 3:**
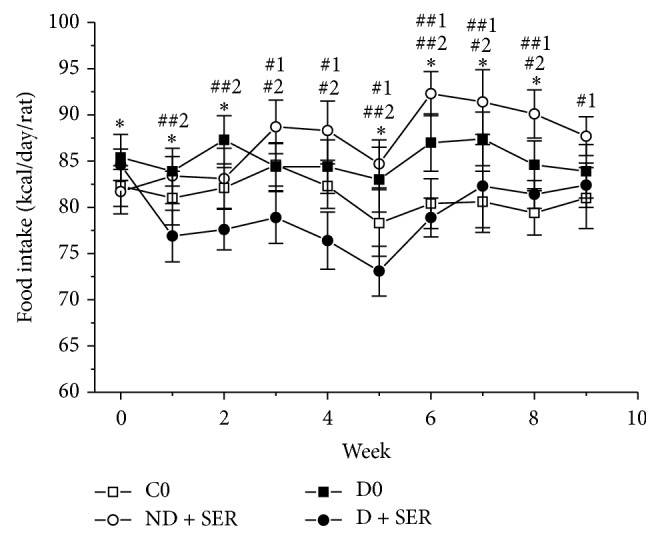
The influence of two-month treatment with intranasally administered serotonin on food intake in diabetic and nondiabetic rats. The data are presented as M ± SD. (*∗*) The difference between Groups C0 and D0 is significant at *P* < 0.05, and (#, ##) the difference between Groups C0 and ND + SER (1) or between Groups D0 and D + SER (2) is significant at *P* < 0.05 and *P* < 0.0001, respectively.

**Figure 4:**
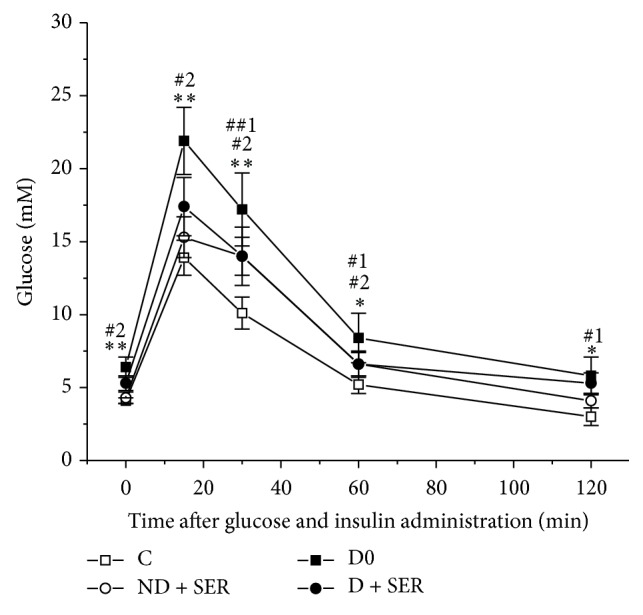
The concentration of plasma glucose in the course of insulin glucose tolerance test in diabetic and nondiabetic rats on the 140th day of experiment. 1—Group C0 (*n* = 6), 2—Group ND + SER (*n* = 8), 3—Group D0 (*n* = 10), and 4—Group D + SER (*n* = 8). The data are presented as M ± SD. (*∗*, *∗∗*) The difference between Groups C0 and D0 and (#, ##) the difference between Groups C0 and ND + SER (1) or between Groups D0 and D + SER (2) are significant at *P* < 0.05 and *P* < 0.0001, respectively.

The food consumption in Groups D and C during the fourth and fifth months of experiment did not differ significantly (Figure 3), but fat intake by diabetic rats was still much higher as compared to the respective control. By the end of experiment, on the 150th day, diabetic rats untreated with IS had weight gain, plus 27% compared with Group C0 (one-way ANOVA, *F*(1; 14) = 85.14,* P *= 2.5 *∗* 10^−7^), and much higher level of glucose, but insulin level did not exceed that in Group C0 (Table 1). According to the results obtained in the course of GTT (on the 147th day) and IGTT (on the 140th day), the rats of the Group D0 had impaired glucose tolerance, insulin resistance, and significantly decreased insulin-induced glucose utilization (Figures 2(c), 2(d), and 4). The calculated value of AUC_0–120_ for glucose concentration curves in GTT in Group D0 exceeded that in Group C0 by 132% (2450 ± 435 versus 1055 ± 80, *P* < 0.0001), and the value for IGTT in diabetic rats exceeded that in control by 67% (1314 ± 192 versus 787 ± 75, *P* < 0.0001). The AUC_30–120_ for insulin concentration curves obtained in GTT in control and diabetic rats differed significantly (132 ± 18 versus 178 ± 25, *P* < 0.05), which demonstrated the development of insulin resistance in Group D0.

In Group D0 the levels of triglycerides, total cholesterol, and LDL cholesterol were 92 (*P* = 1.85 *∗* 10^−5^), 43 (*P *= 8.86 *∗* 10^−4^), and 108% (*P *= 4.35 *∗* 10^−5^) higher than in respective control, while the level of HDL cholesterol did not change (Table 2). The ratio of LDL/HDL cholesterol in diabetic animals was twice higher than in control. Altogether, it confirms the development of abnormalities in lipid metabolism in rats with long-term DM2 induced by HFD and low-dose STZ.

### 3.2. The Influence of Two-Month Treatment of Diabetic and Nondiabetic Rats with Intranasal Serotonin on the Body Weight, Glucose Tolerance and Utilization, and Metabolic Parameters

The two-month treatment of diabetic animals with IS at a daily dose of 20 *μ*g/rat led to a decrease of food intake due to a reduced consumption of dry food (Figure 3) but did not change significantly fat intake. The treatment of diabetic rats with IS decreased the body weight and improved some metabolic parameters. On the 150th day of experiment in Group D + SER plasma level of fasting glucose was much lower than in Group D0, remaining, however, higher than in Group C0 (Table 1). Compared with Group D0, plasma insulin level in Group D + SER did not change significantly.

The IS-treated, unlike untreated, diabetic rats had improved glucose tolerance and increased insulin-induced glucose utilization. In GTT at one- and two-hour points after glucose load, glucose level in Group D + SER was 41 and 57% lower than in Group D0 (Figure 2(c)). 15, 30, and 60 minutes after coadministration of glucose and insulin, glucose level in Group D + SER was much lower than in Group D0, which means that with administration of exogenous insulin the utilization of glucose was accelerated (Figure 4). The AUC_0–120_ for glucose concentration in GTT in Group D + SER was reduced by 32%, as compared to Group D (1672 ± 256 versus 2450 ± 435, *P* < 0.0001), while respective AUC_0–120 _value during IGTT in Group D + SER was reduced by 19% in comparison with untreated diabetic cohort (1068 ± 114 versus 1314 ± 192, *P* < 0.01). The glucose-induced insulin increase in Group D + SER, compared to Group D0, was decreased. The AUC_30–120_ for insulin concentration curve in the case of IS-treated diabetic rats was reduced by 14%, as compared to untreated diabetic animals (153 ± 25 versus 178 ± 25, *P* = 0.056). The shape of the curves of time-dependent insulin secretion in GTT in IS-treated and untreated diabetic rats differed significantly (Figure 2(d)). The obtained data gives evidence that the two-month IS treatment improved insulin sensitivity in animals with the HFD/low-dose STZ model of DM2. In Group D + SER the levels of triglycerides and LDL cholesterol and the ratio of LDL/HDL cholesterol were reduced, compared to untreated diabetic animals (Table 2). The above data shows partial restoration of insulin sensitivity and lipid metabolism in the case of long-term IS treatment of diabetic rats.

In Group ND + SER the food intake was increased, and during the second month of IS treatment the difference was statistically significant compared with the control group (Figure 3), which led to the increase of body weight. The IS treatment of nondiabetic rats decreased insulin sensitivity as it follows from a significant increase of plasma insulin level in response to glucose load (the difference between AUC_30–120_ in Groups C0 and ND + SER is statistically significant at *P* = 0.017) (Figure 2(d)). This treatment also reduced the insulin-induced glucose utilization rate, which was shown using IGTT (*t*-test, *P* < 0.001) (Table 1 and Figure 4). Lipid metabolism in Group ND + SER also changed significantly as compared with control animals. The levels of triglycerides, total cholesterol, LDL cholesterol, and the LDL/HDL cholesterol ratio were significantly increased, and the level of HDL cholesterol reduced (*t*-test, *P* < 0.05). This explains why these data speak in favor of a rather considerable abnormality of carbohydrate and lipid metabolism in the case of increased brain serotonin concentration in healthy animals which, contrary to diabetic animals, have no deficiency of serotonin in the CNS.

### 3.3. The Functional Activity of Adenylyl Cyclase Signaling System and Its Regulation by Hormones in the Hypothalamus of Diabetic Rats

The basal AC activity in the synaptosomal membranes isolated from the hypothalamus of diabetic rats was 26.4 ± 0.8 pmol cAMP/min per mg of membrane protein and did not differ from that in Group C0 (25.1 ± 1.3 pmol cAMP/min per mg of membrane protein). In Group D0 the increase of AC activity over its basal level induced by direct activator of G_s_ proteins nonhydrolysable GTP-analog GppNHp (10^−5 ^M) was much smaller than in Group C0 (42.9 ± 1.2 versus 57.5 ± 1.9 pmol cAMP/min per mg of membrane protein, *P* < 0.05). In diabetic and control animals the corresponding effect of forskolin that directly interacts with catalytic site of AC did not differ significantly (82.3 ± 2.4 versus 79.7 ± 1.9 pmol cAMP/min per mg of membrane protein, *P* > 0.05). Thus, in the hypothalamus of diabetic rats the catalytic activity of AC was preserved, but the AC coupling to G_s_ proteins was decreased.

**Figure 5:**
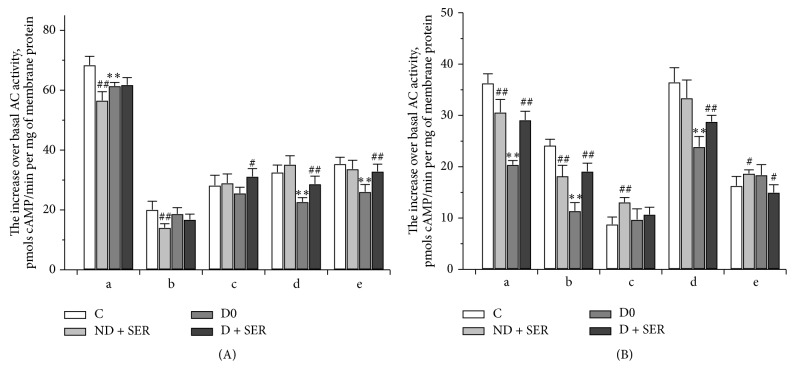
The AC stimulating effects of agonists of monoaminergic (a) and peptidergic (b) receptors in the brain of diabetic and nondiabetic rats and the influence of IS treatment. (A): a—serotonin, b—EMD-386088, c—dopamine, d—norepinephrine, and e—isoproterenol (all were used at a concentration of 10^−5^ M); (B): a—*α*-MSH (10^−7^ M), b—THIQ (10^−7^ M), c—*γ*-MSH (10^−7^ M), d—relaxin (10^−8^ M), and e—PACAP-38 (10^−6^ M). The AC stimulating effect was expressed as hormone-induced increase of AC activity over the basal enzyme activity, pmol cAMP/min per mg of membrane protein. The data are presented as M ± SD. (*∗∗*) The difference between Groups C0 and D0 is significant at *P* < 0.0001, and (#, ##) the differences between Groups C0 and ND + SER or between Groups D0 and D + SER are significant at *P* < 0.05 and *P* < 0.0001, respectively.

In the hypothalamus of diabetic animals the AC stimulating effects of norepinephrine, *β*-agonist isoproterenol, relaxin, *α*-MSH, and selective MC_4_R agonist THIQ were weaker, the corresponding effect of serotonin was lowered, but to a lesser degree, and the effects of dopamine, selective 5-HT_6_R agonist EMD-386088, MC_3_R agonist *γ*-MSH, and PACAP-38 did not change (Figure 5). The decrease of AC stimulation was most significant in the case of *α*-MSH (44%), THIQ (53%), and relaxin (35%). It follows that in the hypothalamus of diabetic rats the signaling cascades involving MC_4_R (but not MC_3_R), relaxin receptors, and *β*-adrenergic receptors (*β*-AR) were reduced.

**Figure 6:**
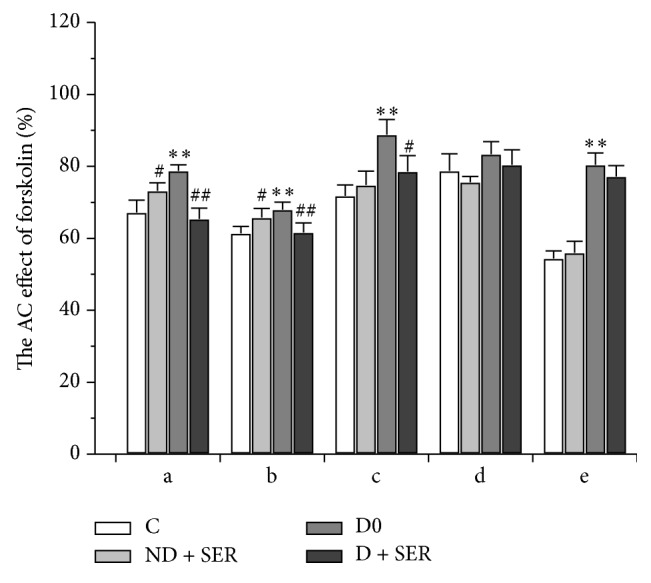
The inhibiting effects of hormones on forskolin-stimulated AC activity in the brain of diabetic and nondiabetic rats with and without IS treatment. a—5-NOT, b—5-MeO-DMT, c—bromocriptine, d—norepinephrine (all were used at a concentration of 10^−5^ M), and e—somatostatin-14 (10^−6^ М). The AC stimulating effect of forskolin (10^−5^ M) in the absence of hormone is taken as 100%. The data are presented as M ± SD. (*∗∗*) The difference between Groups C0 and D0 is significant at *P* < 0.0001, and (#, ##) the difference between Groups C0 and ND + SER or between Groups D0 and D + SER is significant at *P* < 0.05 and *P* < 0.0001, respectively.

The AC inhibiting effects of hormones were evaluated on the basis of their influence on forskolin-stimulated AC activity. In the hypothalamus of diabetic animals AC inhibiting effects of selective 5-HT_1B_R agonist 5-NOT, 5-HT_1/2_R agonist 5-MeO-DMT, DA_2_R agonist bromocriptine, and peptide hormone somatostatin were decreased (Figure 6). Therefore, in the hypothalamus of rats with DM2 there was attenuation of a wide spectrum of G_i_ protein-coupled signaling pathways, suggesting the development of abnormalities in functional activity of G_i_ proteins and their coupling with other signal proteins.

### 3.4. Long-Term Intranasal Serotonin Treatment and Adenylyl Cyclase Signaling in the Hypothalamus of Diabetic and Nondiabetic Rats

The IS treatment of diabetic and nondiabetic rats had no significant influence on the basal as well as GppNHp- and forskolin-stimulated AC activity. In Group D + SER the basal activity of AC was 24.9 ± 1.5 pmol cAMP/min per mg of membrane protein, and GppNHp- and forskolin-induced increase of AC activity reached 45.3 ± 1.3 and 78.1 ± 3.6 pmol cAMP/min per mg of membrane protein, respectively. In IS-treated diabetic rats the AC stimulating effects of norepinephrine, isoproterenol, *α*-MSH, THIQ, and relaxin were partially restored, while the effect of dopamine increased and exceeded that in the other groups of animals. The effect of PACAP-38 was still reduced as compared to Group D0, and the effect of MC_3_R agonist *γ*-MSH in Group D + SER did not differ from that in Groups C0 and D0 (Figure 5). In Group ND + SER the AC effects of *α*-MSH and THIQ were much weaker, while the corresponding effect of *γ*-MSH, on the contrary, increased compared to untreated control animals. This shows that IS restored MC_4_R signaling in the hypothalamus of diabetic rats but led to the weakening of MC_4_R signaling and to the enhancement of MC_3_R signaling in nondiabetic rats. The decrease of the AC stimulating effects of serotonin and 5-HT_6_R agonist EMD-386088 in Group ND + SER indicates that two-month IS treatment led to reduction of G_s_-mediated serotonin signaling (Figure 5).

The AC inhibiting effects of the 5-HT_1_R agonists (5-NOT and 5-MeO-DMT) and DA_2_R agonist bromocriptine impaired in DM2 were partially or completely restored in IS-treated diabetic rats, but the corresponding effect of somatostatin did not change significantly (Figure 6). In Group ND + SER the inhibitory effects of 5-NOT and 5-MeO-DMT on AC activity stimulated by forskolin were decreased as compared to control (*P* < 0.05). The obtained data allow a conclusion that IS administration led to attenuation of G_i_ protein-mediated serotonin signaling in healthy animals and to its restoration in the hypothalamus of diabetic rats.

### 3.5. Adenylyl Cyclase Signaling System and Its Regulation by Hormones and Nonhormonal Activators in the Myocardium of Diabetic Rats: The Influence of Intranasal Serotonin

The basal activity of AC and its increase induced by forskolin (31.1 ± 1.5 and 124.5 ± 3.1 pmol cAMP/min per mg of membrane protein) in the myocardium of diabetic rats did not differ from those in Group C0 (29.2 ± 0.9 and 128.3 ± 2.8 pmol cAMP/min per mg of membrane protein). The increase of AC activity under the action of GppNHp in Group D0 was significantly decreased as compared to control (47.3 ± 1.3 versus 62.5 ± 2.0 pmol cAMP/min per mg of membrane protein, *P* < 0.05).

**Figure 7:**
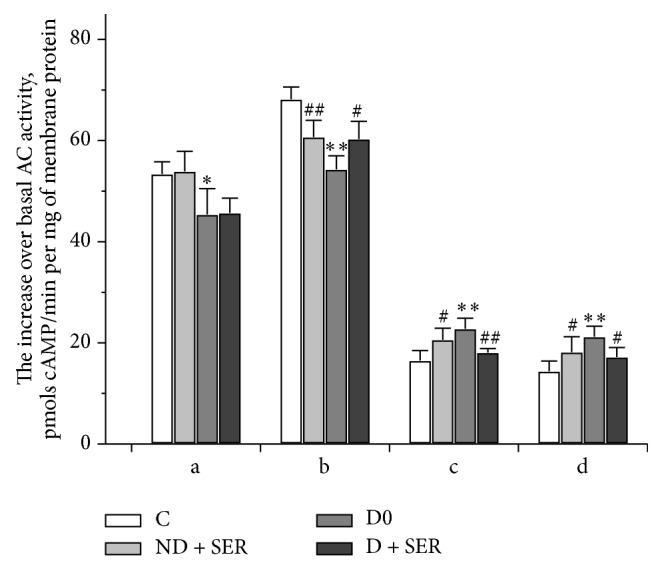
The AC stimulating effects of agonists of adrenergic receptors in the myocardium of diabetic and nondiabetic rats and the influence of IS treatment. a—norepinephrine, b—isoproterenol, c—BRL-37344, and d—CL-316243 (all were used at a concentration of 10^−5^ M). The AC stimulating effect was expressed as hormone-induced increase of AC activity over the basal enzyme activity, pmol cAMP/min per mg of membrane protein. The data are presented as M ± SD. (*∗*, *∗∗*) The difference between Groups C0 and D0 and (#, ##) the difference between Groups C0 and ND + SER or between Groups D0 and D + SER are significant at *P* < 0.05 and *P* < 0.0001, respectively.

In the myocardium of diabetic rats the AC stimulating effects of norepinephrine and isoproterenol, preferably activating *β*_1_- and *β*_2_-AR, were decreased. The corresponding effects of selective *β*_3_-AR agonists BRL-37344 and CL-316243, on the contrary, increased (Figure 7). In animals with DM2 the treatment with IS led to normalization of *β*-AR signaling. In nondiabetic rats the IS treatment led to alterations in the *β*-AR signaling, enhancing the *β*_3_-AR signaling induced by BRL-37344 and CL-316243 and reducing the *β*_1_/*β*_2_-AR signaling induced by isoproterenol. Thus, there were similar changes in *β*_1_/*β*_2_/*β*_3_-AR ratio and in *β*_3_-AR signaling in Groups D0 and ND + SER.

**Figure 8:**
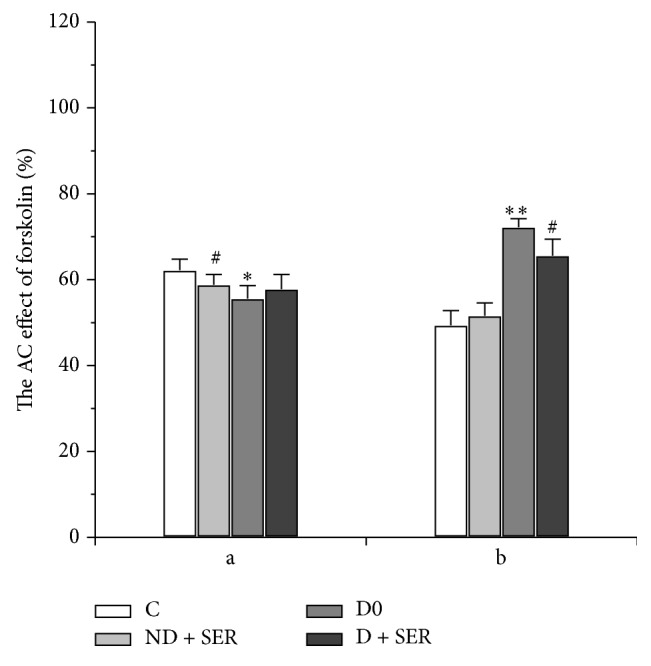
The AC inhibiting effects of norepinephrine and somatostatin in the myocardium of diabetic and nondiabetic rats with and without IS treatment. a—norepinephrine (10^−5^ M) and b—somatostatin-14 (10^−6^ М). The AC stimulating effect of forskolin (10^−5^ M) in the absence of hormone is taken as 100%. The data are presented as M ± SD. (*∗*, *∗∗*) The difference between Groups C and D0 is significant at *P* < 0.05 and *P* < 0.0001, respectively, and (#) the difference between Groups C and ND + SER or between Groups D0 and D + SER is significant at *P* < 0.05.

Finally, in Group D0 the inhibiting effect of somatostatin on forskolin-induced AC activity was significantly reduced but the corresponding effect of norepinephrine, on the contrary, increased compared to Group C0. The treatment of diabetic rats with IS partially restored AC inhibiting effect of somatostatin in diabetic myocardium but had little influence on the same effect of norepinephrine (Figure 8).

## 4. Discussion

Back in the late 1970s it was found that in the brain of rats with the type 1 DM the level of tryptophan, a precursor of serotonin, was markedly reduced [53]. Subsequently, it was established that the concentration of serotonin and 5-hydroxyindole acetic acid (5-HIAA), the metabolite of serotonin, and the conversion of 5-HIAA into serotonin were notably decreased in the brain of rats with long-term and spontaneous DM of the type 1 [54–56]. As for the decrease of serotonin level in the CNS, it was caused mainly by the reduction of tryptophan uptake in the brain and the inhibition of tryptophan-5-hydroxylase-2, the rate-limiting enzyme of serotonin synthesis [53, 56, 57]. It is well-known that insulin lowers plasma levels of aliphatic and aromatic amino acids, such as valine, leucine, isoleucine, phenylalanine, and tyrosine that compete with tryptophan for the transport into the brain. Since in diabetic state the concentration of neutral amino acids has a tendency to increase, it results in the reduction of tryptophan's brain uptake and leads to a deficiency of brain serotonin. There is a lot of evidences that the free tryptophan level and the ratio of free/total tryptophan were decreased in the CNS of patients with DM2 and metabolic syndrome [27, 58–61], as well as with the type 1 DM [62], which has impact on the tryptophan bioavailability in the diabetic brain.

The decrease of serotonin concentration and the changes of serotonin transport and metabolism in the brain led to abnormalities in the serotonin and other neurotransmitter systems, alterations of the number and affinity of 5-HTR, and, as a result, the impairment of serotonin-mediated regulation of energy metabolism and insulin sensitivity in the CNS and the periphery [61, 63, 64]. It should be noted that the DM2-induced changes of serotonin signaling in the CNS are largely dependent on the brain area and are receptor-specific, inducing a wide range of physiological and biochemical responses [65].

As a consequence, the approaches focusing on the increase of brain serotonin level can significantly improve glycemic control and insulin sensitivity. This is confirmed by the results of clinical trials with application of SSRI widely used in the treatment of depression and other neurological disorders. Thus, it was shown that SSRI, fluoxetine in particular, not only improved the psychic state of diabetic patients but also had a rather strong therapeutic effect on DM2 and metabolic syndrome [10, 20, 21, 23, 66]. SSRI normalized the glucose level, reduced blood concentration of glycosylated hemoglobin, caused weight loss, and improved the insulin sensitivity in the CNS and the peripheral tissues and also restored the brain signaling network, which was illustrated by elimination of some DM2-associated neurological dysfunctions. All this gives grounds to say that the increase of brain serotonin level and the restoration of serotoninergic neurotransmission may provide the optimization of metabolic control in diabetic patients, especially in the case of DM2.

In accordance with what has been said above, in this study we demonstrated that two-month IS treatment of rats with HFD/low-dose STZ model of overt DM2 led to the decrease of body weight and improved metabolic parameters, namely, glycemia, insulin resistance, glucose tolerance, and insulin-induced glucose utilization. There was also a decrease of triglycerides and LDL cholesterol levels and of LDL/HDL cholesterol ratio. This positive effect of IS on the metabolic parameters in experimental DM2 is likely to be due to the fact that the long-term administration of serotonin into CNS partially compensates decrease of the brain serotonin level typical of DM2 and metabolic syndrome and restores the serotonin signaling and the other neurotransmitter systems in the CNS. The conditions chosen for our earlier experiments allowed us to demonstrate that long-term administration of IS to female rats with neonatal model of DM improved metabolic parameters and cognitive functions [13].

According to the other authors, the two-week treatment of obese glucose-intolerant mice with selective 5-HT_2C_R agonist BVT.X significantly improved glucose tolerance and reduced the plasma insulin level. The improvements of glucose homeostasis induced by 5-HT_2C_R agonist occurred at its concentration that had no influence on feeding behavior, energy expenditure, locomotor activity, and body weight [22]. To explain our and the other authors' results, it should be taken into account that decrease of the functional activity of G_q_ protein-coupled 5-HT_2C_R in the hypothalamus not only led to hyperphagia and obesity but also considerably reduced insulin sensitivity [67]. This is largely due to the weakening of hypothalamic melanocortin signaling system closely linked to 5-HT_2C_R [22, 68]. 5-HT_2C_R are colocalized with MC_4_R in neurons of the arcuate nucleus of the hypothalamus that are involved in the control of insulin secretion, feeding behavior, and energy expenditure [40, 41], and MC_4_R were shown to be directly involved in the effects of 5-HT_2C_R agonists on insulin sensitivity [22]. 5-HT_1_R can also be involved in the regulation of insulin sensitivity. The activation of 5-HT_1B_R by* m*-chlorophenylpiperazine, a mixed agonist of 5-HT_1B_R and 5-HT_2C_R, was shown to lead to a decrease of appetite in mice lacking 5-HT_2C_R and partially restored insulin sensitivity. This may be the evidence for interaction between the hypothalamic signaling pathways that include 5-HT_1B_R, 5-HT_2C_R, and MC_4_R, all of which are responsible for glucose and insulin tolerance and feeding behavior [68]. The data obtained in the present study on ACSS regulation by agonists of MCR and 5-HTR in the hypothalamus confirms and expands this view. We showed that increase of the brain serotonin level in diabetic rats led to restoration of the AC stimulating effects of MC_4_R agonists and the AC inhibiting effects of 5-HT_1_R agonists dramatically decreased in DM2.

The positive influence of IS on metabolic indices and insulin resistance is largely due to the involvement of serotonin signaling in the regulation of feeding behavior [24–26]. The regulatory effects of serotonin on the appetite and food intake and its significant role in the food-directed motivation are mediated via different types of 5-HTR involved in the regulation of many intracellular cascades and depend on 5-HTR localization in specific brain areas and on functional activity of the other signaling systems. In our experiments, the anorectic effect of centrally administered serotonin is demonstrated as a decrease of body weight and food intake in Group D + SER. This is in agreement with the restoration of hypothalamic MC_4_R signaling, which is responsible for a decrease of food consumption. It was shown by the other authors that the injection of serotonin into the medial hypothalamus of rats reduced food intake elicited by norepinephrine [46]. The injection of the 5-HT_1A_R agonist 8-hydroxy-2-(di-*n*-propylamino)tetralin (8-OH-DPAT) and the 5-HT_2C_R agonist Ro-60-0175 into the paraventricular nucleus, of the 5-HT_1B_R agonist CP-93,129 into the parabrachial nucleus of the pons, and of 8-OH-DPAT into the anterior medial nucleus accumbens caused a significant decrease of food intake and led to the changes of dietary preferences [24–26, 69]. Note that the bilateral infusion of the 5-HT_6_R agonist EMD-386088 into the nucleus accumbens caused the increase of food intake in food-restricted rats as well as in animals on fat/sucrose diet [70]. This data indicates that in the same brain area 5-HT_1_R and 5-HT_6_R regulate the appetitive components of food-directed motivation in different way, and their role in modulating the food consumption is different [71].

In the hypothalamus of diabetic rats IS restored the AC inhibiting effect of DA_2_R agonist bromocriptine significantly reduced in DM2. The brain DA_2_R signaling pathways participate in the control of systemic glucose homeostasis, insulin resistance, feeding behavior, and energy expenditure [72, 73]. The attenuation of DA_2_R signaling in the hypothalamus led to activation of the hypothalamic-pituitary-adrenal (HPA) axis and stimulated the release of cortisol, increased the activity of the sympathetic nervous system, and enhanced the latter's influence on the liver, adipose tissue, and the cardiovascular system [19, 74, 75]. The long-term activation of the sympathetic nervous system in adipose tissue induced the alterations of lipid metabolism, increased the secretion of proinflammatory factors, and, eventually, provoked insulin resistance [76]. This was the starting point for a wide application of bromocriptine and its pharmacological analogs to correct insulin resistance and energy metabolism in DM2 and metabolic syndrome [16–18, 77]. Hence, IS-induced restoration of the DA_2_R signaling in the hypothalamus can be regarded as one of the mechanisms of positive influence of central serotonin on glucose and lipid homeostasis.

In the hypothalamus of diabetic rats the IS treatment also restored AC stimulating pathways regulated by *β*-AR agonists and relaxin, which gives reasonable grounds to suppose that the long-term IS treatment ameliorates blood circulation in the CNS. Morphological and biochemical abnormalities in the cerebral microvessels often induce microcirculatory disorders in the hypothalamus and the other brain areas, leading to hypoxia, neuronal death, and neurodegenerative processes. It was shown that in cerebral microvessels of diabetic rats both the number of *β*-AR and the AC stimulating effect of isoproterenol are reduced [78, 79].

Quite different results were obtained in long-term IS treatment of nondiabetic rats. This treatment led to metabolic changes such as increased body weight and decreased glucose utilization. There was also alteration in hypothalamic signaling, such as attenuation of MC_4_R-mediated signaling and G_s_ protein- and G_i_ protein-coupled serotonin signaling, and increase of MC_3_R-mediated signaling. It seems likely that a long lasting increase of the brain serotonin gives rise to the resistance to it; this leads to the weakening of serotonin signaling in the hypothalamus and MC_4_R signaling functionally associated with it. An increase in the functional activity of MC_3_R is probably due to the compensatory changes in the melanocortin signaling on the background of MC_4_R deficiency. The information is available showing that the three-week treatment of rats with fluoxetine increased serotonin concentration in the rat brain and led to a decrease of brain 5-HT_1A_R response to the 5-HT_1A_R agonist 8-OH-DPAT and to an increase of 5-HT_2A/2C_R response to the 5-HT_2_R agonist 1-(2,5-dimethoxy-4-iodophenyl)-2-aminopropane [80]. The decrease of functional activity of 5-HT_1A_R and the desensitization of hypothalamic postsynaptic 5-HT_1A_R signaling was also observed after 15-day treatment of cycling female rats with fluoxetine [81]. The consequences of prolonged elevation of serotonin level in the brain must be given proper attention in the treatment of non-DM pathology, such as depression and neurological dysfunctions, with SSRI and the agonists of 5-HTR [82, 83]. The negative influence of the long-term increase of brain serotonin level on the metabolic status of nondiabetic rats can be caused by several factors. Along with the dysregulation of integrative signaling network in the hypothalamus and some other brain areas, leading to insulin resistance and neuroendocrine system impairment, there are disturbances in the serotonin-mediated response to glucoprivation in the hypothalamus, hyperactivation of the HPA axis, and disruption of the circadian time rhythm.

As is known, hypothalamic serotonin enhances the counterregulatory response to hypoglycemic and glucoprivic stimuli and in a glucose-dependent manner controls feeding behavior. Insulin-induced hypoglycemia stimulates the release of serotonin in widespread forebrain regions, including the perifornical, ventromedial, and paraventricular hypothalamus, and induces food consumption. It was shown that 5-thioglucose-induced glucoprivation in the perifornical hypothalamus increased serotonin level and led to 4-fold increase of food intake and 30-fold increase of epinephrine release in the adrenal medulla [44]. Since in the paraventricular nucleus of hypothalamus the serotonin neurons project to and activate the corticotropin-releasing factor neurons, the increase of serotonin level resulted in the activation of the HPA axis and in the increase of the levels of ACTH and corticosterone [84–86]. Hypothalamic serotonin regulated the HPA axis preferably via 5-HT_2C_R which are coexpressed with the corticotropin-releasing factor neurons in the paraventricular nucleus [87]. In Sprague-Dawley rats the serotonin-mediated activation of HPA axis exhibited a significant increase of body weight, of the abdominal circumference and the abdominal white adipose tissue mass, and in addition led to impaired glucose tolerance, fasting hyperglycemia, hyperinsulinemia, and significantly decreased expression of insulin receptor in the hypothalamus, indicating the development of central insulin resistance [88]. Some of these metabolic dysregulations correspond to those identified by us in Group ND + SER. It may well be that the cause of metabolic abnormalities induced by IS administration into nondiabetic animals is a change of circadian rhythm, which had a direct influence on the functions of HPA axis and provoked disturbances in synchronization of the release of serotonin and other neurotransmitters, dopamine in particular [89, 90].

The influence of the IS treatment on the AC signaling system sensitive to *β*-AR agonists in the myocardium was also dependent on the metabolic status of the animals and was significantly different in control and diabetic rats. In DM2 animals, the AR signaling which is implemented through *β*_1_/*β*_2_-AR was decreased, while the AR signaling realized via *β*_3_-AR was, on the contrary, enhanced. This, in our view, can be provoked by different ratio of the types of *β*-AR in the diabetic myocardium. The treatment of diabetic rats with IS partially restored *β*_1_/*β*_2_/*β*_3_-AR ratio, increasing the AC effects of *β*_1_/*β*_2_-agonist isoproterenol and reducing respective effects of *β*_3_-agonists BRL-37344 and CL-316243. However, in the case of nondiabetic rats the same treatment resulted, on the contrary, in an imbalance in favor of the *β*_3_-AR signaling pathways, like it was with the myocardium of untreated diabetic rats. These data are consistent with those of other authors which showed that increasing the level of serotonin in the brain due to the long-term treatment of experimental animals with fluoxetine resulted in the increase of norepinephrine level and dysfunctions of the cardiovascular system [91, 92]. The increase of catecholamine level is a result of serotonin-induced activation of the HPA axis mediated via the sympathetic nervous system or via the hypothalamic secretion of the corticotropin-releasing factor [44].

The authors studying patients with DM2 and metabolic syndrome, as well as experimental animals with the type 1 DM, demonstrated that AR signaling realized through *β*_1_-AR was significantly reduced, while the *β*_3_-AR signaling increased [93–95]. The enhancement of functional activity of *β*_3_-AR in diabetic myocardium may have been due to two compensatory mechanisms. The first involved AC stimulating effect of catecholamines that was decreased under diabetic conditions when the plasma level of catecholamines significantly increased [96]. It should be noted that, generally, AC stimulatory effect of catecholamines is realized through *β*_1_- and *β*_2_-AR. The second mechanism was a compensatory reinforcement of NO-synthase signaling pathway, the target of *β*_3_-AR agonists, reduced in DM2 [95]. In the present study, the AC stimulating effect of GppNHp was decreased, which can also be interpreted as a compensatory response providing conditions for hyperactivation of AC signaling system by catecholamines to be reduced in the diabetic myocardium.

A positive impact of the elevated level of brain serotonin on AC signaling in the diabetic myocardium gives evidence for direct relationship between the alterations in the brain signaling, serotonin signaling in particular, and the functions of the cardiovascular system in DM2 [97–99]. There are other causes of cardiovascular disorders in DM2. These are abnormalities of carbohydrate and lipid metabolism, lipotoxicity, oxidative stress, insulin resistance, and increased levels of proinflammatory factors, all of which lead to damage of the vascular endothelial cells, activation of inflammatory processes, and formation of atherosclerotic plaques [100]. At least some of these factors also have the central genesis, which is related to the impairment of CNS signaling.

Thus, the IS treatment of diabetic animals with brain serotonin deficiency gives some improvement of glucose tolerance, insulin sensitivity, and hormonal signaling in the hypothalamus and myocardium, while in nondiabetic animals with normal level of brain serotonin it gives, on the contrary, a negative impact. The functional response to an increase of brain serotonin level depends, to a large extent, on the state of the hypothalamus integrative system and the neuroendocrine system and varies significantly in the metabolic disorders, such as DM and obesity, and in different physiological states, including pregnancy. This is illustrated by the results of the study, wherein the long-term stimulation of nonpregnant ewes by intracerebroventricular infusion of fluoxetine reduced food intake but had no influence on the expression of proopiomelanocortin mRNA in the hypothalamus. However, such infusion into pregnant ewes decreased the production of proopiomelanocortin but did not change significantly the feeding behavior [101]. It should be taken into account that the acute or short-term treatment of animals with centrally administered SSRI, serotonin and 5-HTR agonists, led to the results which differed considerably from those obtained in the case of chronic treatment. In our experiments the IS treatment covered the period of 60 days, that is, long enough to study the effect of chronic elevation of central serotonin level on metabolic parameters and hormonal signaling in diabetic and nondiabetic rats. The scheme of treatment we followed is in good agreement with real situation when diabetic and nondiabetic patients are treated for a long time with antidepressants acting on the brain serotonin system.

## 5. Conclusion

We showed that a long-term (two-month) treatment of diabetic rats with intranasally administered serotonin improved glucose tolerance and utilization, insulin sensitivity, and lipid metabolism. These positive effects of IS are accounted for, to a large extent, by the restoration of integrative signaling network of the brain due to normalization of brain serotonin level which is decreased in DM2. As a consequence, the sensitivity of the hypothalamic AC signaling system to monoamines and peptide hormones was partially restored. The treatment with IS also restored the balance between adrenergic signaling pathways in the myocardium of diabetic rats, which in diabetic pathology was shifted toward the *β*_3_-AR signaling. The data is available indicating that IS and other substances capable of elevating serotonin level in the CNS are effective in treating both DM2- and DM-associated disorders of the nervous and cardiovascular systems. At the same time, the two-month treatment of nondiabetic rodents with IS led to metabolic and hormonal alterations due to long-term hyperactivation of the brain serotonin system, and this should be taken in consideration in the development of therapeutic approaches involving long-term treatment of nondiabetic patients with drugs that increase the brain serotonin level.

## Figures and Tables

**Table 1:** The body weight and the plasma level of fasting glucose and insulin in control and diabetic rats and the influence of the treatment with IS.

	Start point of the experiment, 0 day	60th day (two-month HFD)	90th day (before IS treatment)	150th day (before decapitation)
	Group C (*n* = 14)			Group C0 (*n* = 6)

Weight, g	181 ± 8	298 ± 15	337 ± 14	359 ± 22
Glucose, mM	4.0 ± 0.2	4.0 ± 0.3	4.3 ± 0.3	3.8 ± 0.3
Insulin, ng/mL	ND	0.56 ± 0.17	ND	0.67 ± 0.15

				Group ND + SER (*n* = 8)

Weight, g	—			419 ± 29^#^
Glucose, mM	—			4.5 ± 0.3^#^
Insulin, ng/mL	—			0.84 ± 0.21

	Group D (*n* = 18)			Group D0 (*n* = 10)

Weight, g	186 ± 7	372 ± 18^*∗∗*^	410 ± 20^*∗∗*^	457 ± 19^*∗∗*^
Glucose, mM	3.9 ± 0.2	5.5 ± 0.5^*∗∗*^	7.8 ± 1.0^*∗∗*^	6.9 ± 0.8^*∗∗*^
Insulin, ng/mL	ND	0.74 ± 0.15^*∗*^	ND	0.76 ± 0.16

	—			Group D + SER (*n* = 8)

Weight, g	—			429 ± 25^#^
Glucose, mM	—			5.2 ± 0.5^##^
Insulin, ng/mL	—			0.73 ± 0.11

*Note*. All values are presented as M ± SD. (*∗*, *∗∗*) The difference between Groups C and D and between Groups C0 and D0 and (#, ##) the difference between Groups C0 and ND + SER or between Groups D0 and D + SER are significant at *P* < 0.05 and *P* < 0.0001, respectively.

**Table 2:** The triglycerides, total cholesterol, LDL and HDL cholesterol levels, and ratio of LDL/HDL cholesterol in diabetic and control rats on the 150th day of experiment and the influence of the treatment with IS.

	Triglycerides, mM	Total cholesterol, mM	HDL cholesterol, mM	LDL cholesterol, mM	LDL cholesterol/HDL cholesterol ratio
C0 (*n* = 6)	0.85 ± 0.13	4.49 ± 0.35	2.89 ± 0.14	1.42 ± 0.17	0.49 ± 0.04
ND + SER (*n* = 8)	1.12 ± 0.16^#^	5.02 ± 0.30^#^	2.61 ± 0.18^#^	1.74 ± 0.16^#^	0.67 ± 0.04^##^
D0 (*n* = 10)	1.63 ± 0.28^*∗∗*^	6.42 ± 1.08^*∗*^	2.86 ± 0.16	2.96 ± 0.63^*∗∗*^	1.03 ± 0.20^*∗∗*^
D + SER (*n* = 8)	1.35 ± 0.14^#^	5.65 ± 0.56	3.03 ± 0.23	2.33 ± 0.34^#^	0.77 ± 0.11^#^

*Note*. The data are presented as M ± SD. (*∗*, *∗∗*) The difference between Groups C0 and D0 and (#, ##) the difference between Groups C0 and ND + SER or between Groups D0 and D + SER are significant at *P* < 0.05 and *P* < 0.0001, respectively.

## Abbreviations

AC: Adenylyl cyclase
ACSS: Adenylyl cyclase signaling system
AR: Adrenergic receptor
CNS: Central nervous system
DAR: Dopamine receptor
DM2: Type 2 diabetes mellitus
G_s_ and G_i_ proteins: Heterotrimeric G proteins of the stimulatory and inhibitory types, respectively
GTT: Glucose tolerance test
HFD: High-fat diet
HPA axis: Hypothalamic-pituitary-adrenal axis
5-HIAA: 5-Hydroxyindole acetic acid
5-HTR: 5-Hydroxytryptamine receptor
IGTT: Insulin glucose tolerance test
IS: Intranasal serotonin
LDL and HDL cholesterols: Low-density lipoprotein and high-density lipoprotein cholesterols
MCR: Melanocortin receptor
MSH: Melanocyte-stimulating hormone
8-OH-DPAT: 8-Hydroxy-2-(di-*n*-propylamino)tetralin
PACAP-38: Pituitary adenylyl cyclase-activating polypeptide-38
SSRI: Selective serotonin reuptake inhibitor
STZ: Streptozotocin
BRL-37344: (±)-(*R* ^*∗*^, *R* ^*∗*^)-[4-[2-[[2-(3-Chlorophenyl)-2-hydroxyethyl]amino]propyl]phenoxy]acetic acid sodium hydrate
CL-316243: 5-[(2R)-2-[[(2R)-2-(3-Chlorophenyl)-2-hydroxyethyl]amino]propyl]-1,3-benzodioxole-2,2-dicarboxylate disodium hydrate
EMD-386088: 5-Chloro-2-methyl-3-(1,2,3,6-tetrahydro-4-pyridinyl)-1H-indole
5-MeO-DMT: 5-Methoxy-*N,N*-dimethyltryptamine
GppNHp: *β*,*γ*-Imidoguanosine-5′-triphosphate
5-NOT: 5-Nonyloxytryptamine
THIQ: N-[(1R)-1-[(4-Chlorophenyl)methyl]-2-[4-cyclohexyl-4-(1H-1,2,4-trazol-1-ylmethyl)-1-piperidinyl]-2-oxoethyl]-1,2,3,4-tetrahydro-3-isoquinolinecarboxamide
AUC: Area under the curve.
